# Knowledge and Attitudes towards Complementary and Alternative Medicine among Senior Medical Students in King Abdulaziz University, Saudi Arabia

**DOI:** 10.1155/2016/9370721

**Published:** 2016-03-14

**Authors:** Sami H. Alzahrani, Jamil Bashawri, Emad M. Salawati, Marwan A. Bakarman

**Affiliations:** ^1^Family and Community Medicine Department, Faculty of Medicine, King Abdulaziz University, P.O. Box 80205, Jeddah 21589, Saudi Arabia; ^2^Family and Community Medicine Department, Rabigh Faculty of Medicine, King Abdulaziz University, P.O. Box 80205, Jeddah 21589, Saudi Arabia

## Abstract

*Objectives*. This study assessed the knowledge and attitudes regarding complementary and alternative medicine (CAM) in medical students in Saudi Arabia. Furthermore, it evaluated their views on the incorporation of CAM in their medical syllabus.* Methods*. The study was conducted by selecting a cross-sectional sample of senior medical students in the Faculty of Medicine. A validated and reliable self-administered questionnaire was used to explore the knowledge, attitude, and benefits of CAM. It was distributed to a sample of 273 students.* Results*. The study included 242 students, making the response rate 88.6%. Only two-thirds of students (62.4%) were aware of acupuncture principles and only 17.4% recognized that chiropractic is associated with pain management. The knowledge of common herbs such as St. John's Wort,* Echinacea*, and* Ginkgo biloba* was limited among the students. Older students had a positive CAM attitude compared to younger students (*p* = 0.027).* Conclusion*. Students attitudes toward CAM learning were encouraging regardless of their limited knowledge on the subject. A high percentage of students agreed that CAM in combination with conventional therapy is beneficial in treating unusual cases, but the choice of CAM should be based on evidence. Furthermore, medical students are still reluctant to have CAM practitioners in their referral network.

## 1. Introduction

It is evident from the history of healing through medicine that traditional therapies have been successfully used for treatment alongside the use of conventional medicine practices. The advancements in the field of conventional therapy have failed to overshadow the beneficial effects produced by traditional medicine (TM) among patients. The scope of TM is widely spread and is extended to various approaches of treatment, including materials extracted from plant herbs, animals, and mineral deposits. Moreover, spiritual remedies and physiotherapy treatment are also included in the domain of traditional medicine. These traditional therapies are included alone or in combination for the purpose of the diagnosis, treatment, and prevention of illness [1].

Traditional medicine is known as “complementary and alternative medicine” (CAM) in some countries. The World Health Organization (WHO) defines TM as “the knowledge, skills, and practices based on the theories, beliefs, and experiences indigenous to different cultures, used in the maintenance of health and in the prevention, diagnosis, improvement, or treatment of physical and mental illness” [2]. CAM is defined by the WHO as “a broad set of healthcare practices that are not part of that country's own tradition and are not integrated into the dominant healthcare system” [2].

In present practices, it is thought that all the diagnostic and treatment therapies that lie outside the principles of conventional therapy come under the discipline of CAM [3]. Therefore, TM and CAM are less widely acceptable around the world than the acceptable conventional therapies. CAM therapies that are projected as safe and effective universally are also adopted in the conventional system [4].

It should be noted that 80% of sick individuals, particularly in developing countries, depend more on complementary therapies rather than conventional healthcare, whereas the percentage using CAM therapy has declined to half among the population in industrialized countries [5]. It has always been “invisible mainstream” of treatment for millions of patients around the world [6].

The WHO [7] reported that the use of CAM has increased around the world. However, CAM is also being used as an additional therapy along with the conventional therapies for a long period of time [8]. The use of multiple therapeutic modes in addition to conventional medicine therapy is called “medical pluralism.” These healthcare modalities are consumed by millions of beneficiaries irrespective of the unofficial status of alternative medicine [9].

The popularity of CAM among healthcare professionals and the population could be observed from its increased use in modern-day healthcare practices. In spite of the increasing use of alternative therapies, it has not been included in the medical curricula of many medical universities around the world [10]. The use of CAM in recent treatment approaches has increased rapidly and is also being referred to by many healthcare professionals [11, 12]. The wall of conflicting opinions in the choice between conventional and alternative medicine seems to be insurmountable.

It is advisable to develop a strong knowledge base among medical students regarding the recommendations and uses of CAM. It is therefore suggested to assess students' awareness level and willingness to incorporate CAM therapies in the integrative medicine curriculum.

There is a need to change the attitude of healthcare professionals regarding the use of complementary medicine on their patients due to the mere fact of its increasing use around the world. This awareness and knowledge attainment of CAM therapies will enable healthcare professionals to select the best and appropriate complementary medicine for their patients. The use of CAM in developing countries is more frequent than in developed countries. This is most likely due to various influencing factors, such as the personal, religious, and spiritual beliefs of people in Saudi Arabia. The objective of this study is to assess the awareness of senior medical students in King Abdulaziz University regarding CAM and their willingness to be trained in this discipline for their professional future.

## 2. Materials and Methods

The participants for this study were randomly selected from students of the Faculty of Medicine, King Abdulaziz University. During the study period, there were 933 medical students, 457 males and 476 females, in the clinical phase of the curriculum, that is, 4th year (183 males and 186 females), 5th year (123 males and 117 females), and 6th year (151 males and 173 females).

The Raosoft software package was used to calculate the sample size, and the single proportion method was used [13]. By assuming that 50% of senior medical students will have sufficient knowledge and a positive attitude toward CAM, the required sample size was 273 students at a 95% confidence interval and a 5% margin of error. Proportional allocation of the students was performed using the stratified sampling technique as follows: 40% of the sample size was taken from 4th-year, 25% from 5th-year, and 35% from 6th-year academic students. The questionnaire was previously used in an Australian study involving 800 students in similar academic years [14] (permission was taken to use the questionnaire). The questionnaire included personal biodata and questions to assess knowledge about alternative medicine. The students' awareness about the beneficial effects of the seven CAM modalities and four herbs was also sought.

After obtaining the acceptance of the Faculty of Medicine administration and clearance from the KAU Research and Ethics Committee, students' consent was obtained and documented. The study commenced at the beginning of October 2015.

The questionnaires were distributed to the students at the end of their academic sessions throughout the month of October 2015 and were returned to a predetermined desk in the Family Medicine Department at the end of each day, where one of the researchers was available to clarify questions.

Data were entered and analyzed using the Statistical Package for the Social Sciences (SPSS) version 20. The percentage score was calculated by adding the responses of knowledge and attitude items for each student, and then the Mann-Whitney *U* test was applied to compare the median percentage scores by age and gender; the Kruskal Wallis *H*-test was applied to compare median percentage scores by academic year.

## 3. Results

A total of 242 medical students out of 273 completed the questionnaires, for a response rate of 88.6%. Their age range was 22–28 years with a mean of 22.6 ± 1.2. Just over half (51.7%) were males. More than one-third (39.7%) were recruited from the 4th year, 35.5% were selected from the 6th academic year, and the rest were from the 5th year.

As illustrated in Table 1, just over two-thirds of the senior medical students (68.2%) had never heard of feverfew, and more than half had never heard about homeopathy (55%), with similar results for megavitamin therapy (54.1%). Almost one-third of them were unaware of magnetic therapy (38.5%) and ginseng therapeutic use (33.3%).

**Figure 1:**
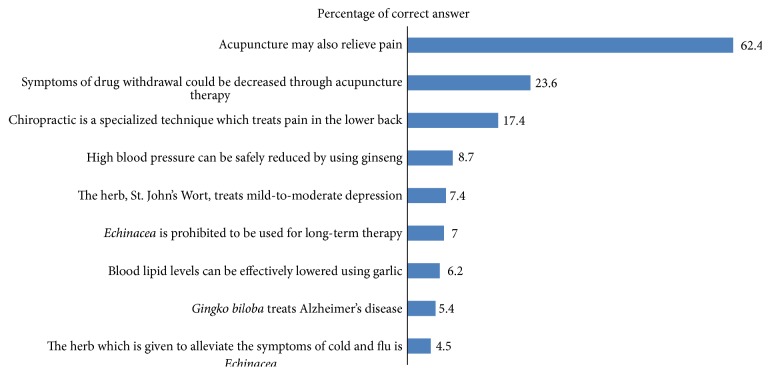
Responses of medical students regarding certain CAM modalities of treatment.

Almost two-thirds of the students (62.4%) knew that acupuncture was considered one of the CAM modalities of treatment, and only 17.4% recognized the role of chiropractic in management of pain. The majority of the students had no knowledge of the uses and side effects of St. John's Wort,* Echinacea*, and* Ginkgo biloba* herbs (Figure 1).

From Table 2, it is evident that female students had greater CAM knowledge than male students (mean ranks: 135.5, *p* = 0.003). Students' age and academic year were not significantly associated with CAM knowledge.

A large proportion (79.8%) of students reported that massage is effective. Almost two-thirds of them reported that ginger (63.2%) and garlic (61.6%) are effective. Acupuncture and herbal medicine were described as effective by 55.8% and 57.9% of them, respectively (Table 3).

In Table 4, we observe that most of the students (75.2%) agreed that physicians should be consulted before using alternative therapy. Almost two-thirds of them (64.5%) stated that conventional medicine can benefit from CAM. More than half of them agreed that CAM therapies should not be used unless they are tested for efficacy (59.1%) and that further clinical care should include only CAM therapies that have produced the best results (57%). It is suggested that healthcare professionals should possess knowledge about CAM to provide accurate advice to their patients (51.6%), and CAM-related knowledge is essential to practicing as a health professional (50.8%).

It is evident from Table 5 that older students (>22 years old) had a positive CAM attitude than younger students (mean ranks were 130.2 and 110.3, *p* = 0.027). Similarly, those in the 6th academic year had a significantly more positive attitude toward CAM than those in 5th and 4th academic years (mean ranks were 142.3 versus 123.4 and 101.7, resp., *p* < 0.001). Students' gender was not significantly associated with their CAM attitude.

## 4. Discussion

CAM is gaining popularity rapidly in the field of medical sciences and is now considered an important branch of the healthcare system. Our study showed consistency with the findings of previous studies [15–18] and identified that medical students have exposure to limited knowledge of CAM during their medical studies.

In the current study, the students had awareness of massaging techniques, herbal medicine, and spiritual healing as forms of CAM therapy. In a study conducted in Singapore, the medical students' results were in contrast to our findings, as they were more knowledgeable about acupuncture, yoga, and homeopathy as techniques of alternative therapy [15]. In Ireland, students were more attracted to the spirituality and massage modalities of CAM [17].

Most of the students in the current study had minimal knowledge about the modality of acupuncture; two-thirds of them were only aware of the principles of acupuncture. This could be attributed to the fact that CAM practitioners are primarily visited for the treatment of acupuncture [19]. A good number of students have a positive attitude toward learning CAM and would welcome its inclusion in their medical curriculum, despite their poor knowledge on the subject. However, students have shown a more positive attitude toward massage, herbal medicine, and spiritual healing modalities, which could be perceived as their awareness about these modalities due to the high popularity of these therapies in the Saudi population [20].

In line with other studies [17, 21], there was no preference of gender regarding the learning of CAM, although females were more knowledgeable than males. It is encouraging that senior academic students showed acceptance toward CAM learning. The majority of the medical students in our study agreed that having knowledge of CAM is important for their professional careers, although they were reluctant to incorporate the CAM courses into their medical curriculum. This was a less welcoming approach for the future of CAM in Saudi Arabia compared to other countries [15]. To promote the learning of CAM, high-quality content that is easier to learn should be incorporated into medical courses, as suggested by Gaster et al. [22]. Student learning could be enhanced by a combination of lectures and direct shadowing. Three-week elective training of CAM was provided during clinical rotations in a US institution, which contained training and lecture sessions. These sessions have proven to be highly significant for improving the skills and attitudes of students [23]. Moreover, the use of evidence-based knowledge to teach alternative therapies is suggested [24].

A cross-sectional study was conducted among Turkish medical students to determine their interest in CAM. Their views about the inclusion of specific CAM training during their residency were also estimated. The findings showed that 81.2% of the students were well acquainted with herbal treatment, 80.8% knew about acupuncture, and 78.8% regarded hypnosis as a part of CAM treatment. A gender-based view of the medical students revealed that female students more often identified the need for meditation and the use of herbs to aid medical treatment. Hence, it is observed that female medical students exhibit a more positive attitude toward the inclusion of CAM in their syllabi than their male counterparts. It was evident from the research findings that medical students in Turkey were more knowledgeable about CAM treatment. Furthermore, they were more likely to undergo training in CAM and recommend complementary medicine to their patients on the basis of patients' conditions [25].

A survey conducted in a medical school in the United States with data obtained from 263 medical students regarding their familiarity with CAM indicated that students' interest in complementary medicine gradually faded as they progressed from their 1st to their 3rd year of medical school. The 1st-year students were more likely to use CAM for their personal needs than medical students in their 3rd year. Moreover, the 3rd-year students exhibited a less favorable attitude toward recommending CAM in their medical practice. It is therefore suggested that students' willingness to recommend complementary medicine to their patients should be enhanced when the conventional treatment strategies failed. It is possible, by the collection of research-based evidence, to assess the safety and effectiveness of complementary medicine. Students should be taught about the benefits and harmful effects of various CAM modalities in light of evidence-based practice so that these complementary treatments could be effectively incorporated into the integrative medicine learning curriculum [26].

In conclusion, the results in our study showed that students' attitudes toward CAM learning were encouraging, regardless of their limited knowledge on the subject. A high percentage of students agreed that alternative medicine in combination with conventional therapy is beneficial in treating unusual cases, but the choice of CAM therapy should be based on evidence. Furthermore, irrespective of the wider use of CAM, medical students are still reluctant to have CAM practitioners in their referral network.

The effectiveness of herbal medicine, spiritual healing, and massage for long-term treatment was generally low according to the opinions of our Saudi medical students. This could be attributed to the fact that safety and efficacy knowledge of these modalities among the students is inadequate and they are reluctant to recommend CAM therapy to their patients. However, most of the students agreed that healthcare practitioners should be aware of their patients' previous or current use of CAM during their history-taking process.

## Figures and Tables

**Table 1:** Knowledge of complementary and alternative medicine among senior medical students.

CAM modalities	Good knowledge	Minimum knowledge	Aware of CAM modalities	No awareness
	*N* (%)	*N* (%)	*N* (%)	*N* (%)
Treatment/technique				
Acupuncture	21 (8.7)	114 (47.1)	80 (33.1)	27 (11.1)
Massage	41 (16.9)	135 (55.8)	57 (23.6)	9 (3.7)
Homeopathy	14 (5.8)	46 (19.0)	49 (20.2)	133 (55.0)
Herbal medicine	35 (14.5)	117 (48.3)	71 (29.3)	19 (7.9)
Megavitamin therapy	9 (3.7)	42 (17.4)	60 (24.8)	131 (54.1)
Spiritual healing	32 (13.2)	68 (28.1)	92 (38.0)	50 (20.7)
Magnetic therapy	3 (1.2)	52 (21.5)	94 (38.8)	93 (38.5)
Herbs				
Feverfew	3 (1.2)	31 (12.8)	43 (17.8)	165 (68.2)
Ginger	57 (23.6)	106 (43.8)	60 (24.8)	19 (7.9)
Garlic	65 (26.9)	106 (43.8)	54 (22.3)	17 (7.0)
Ginseng	26 (10.7)	49 (20.2)	87 (36.0)	80 (33.1)

**Table 2:** Senior medical student's knowledge of CAM.

Students' characteristics	CAM knowledge percentage score			*p* value
	Median	IQR	Mean rank	
Age (years)				
≤22 (*n* = 106)	38.1	31.0–45.8	129.7	0.106
>22 (*n* = 136)	35.7	28.6–40.5	115.1	
Gender				
Males (*n* = 125)	33.3	26.2–40.5	108.4	0.003
Females (*n* = 117)	38.1	31.0–45.2	135.5	
Academic year				
4th (*n* = 96)	36.9	31–45.2	125.2	0.764
5th (*n* = 60)	35.7	26.8–44.6	121.3	
6th (*n* = 86)	35.7	28.6–42.9	117.6	

**Table 3:** Senior medical students' opinion regarding effectiveness of complementary and alternative medicine modalities.

Effectiveness of CAM modalities	Not effective	Not sure of effectiveness	Effective
	*N* (%)	*N* (%)	*N* (%)
Treatment/technique			
Acupuncture	11 (4.54)	96 (39.66)	135 (55.8)
Massage	5 (2.06)	44 (18.18)	193 (79.76)
Homeopathy	19 (7.85)	181 (74.79)	42 (17.36)
Herbal medicine	15 (6.19)	87 (35.95)	140 (57.86)
Megavitamin therapy	23 (9.5)	181 (74.79)	38 (15.71)
Spiritual healing	23 (9.5)	104 (42.97)	115 (47.71)
Magnetic therapy	23 (9.5)	188 (77.68)	31 (12.82)
Herbs			
Feverfew	10 (4.13)	205 (84.71)	27 (11.16)
Ginger	3 (1.23)	86 (35.53)	153 (63.24)
Garlic	8 (3.3)	85 (35.12)	149 (61.58)
Ginseng	7 (2.89)	171 (70.66)	64 (26.45)

**Table 4:** Attitude of senior medical students towards different concepts of CAM.

Statements	Strongly agree	Agree	Neutral	Disagree	Strongly disagree
	*N* (%)	*N* (%)	*N* (%)	*N* (%)	*N* (%)
The ideas and methods applied in CAM could be beneficial for conventional therapies	22 (9.1)	134 (55.4)	70 (28.9)	10 (4.1)	6 (2.5)
Patients should inform/consult their doctors about their use of CAM	50 (20.66)	132 (54.54)	45 (18.6)	10 (4.13)	5 (2.07)
The CAM therapy produced significant effects with placebo testing	19 (7.9)	86 (35.5)	94 (38.8)	40 (16.5)	3 (1.2)
The CAM therapies that are not scientifically tested should not be used to discourage the harmful aspects of CAM	67 (27.7)	76 (31.4)	67 (27.7)	23 (9.5)	9 (3.7)
Clinical care should be a mixture of conventional and complementary medicine to ensure the best results	54 (22.3)	84 (34.7)	84 (34.7)	18 (7.4)	2 (0.8)
Healthcare professionals should be competent enough to advise their patients on the best available CAM method relevant to their treatment	47 (19.4)	78 (32.2)	79 (32.6)	30 (12.4)	8 (3.3)
The spiritual and religious beliefs of students influence their ability to use CAM methods in their professional practice	41 (16.9)	59 (24.4)	90 (37.2)	41 (16.9)	11 (4.5)
CAM knowledge is necessary for the career of students as a future practicing healthcare professional	46 (19.0)	77 (31.8)	77 (31.8)	33 (13.6)	9 (3.7)
Patients should be instructed to consult their doctors before taking additional CAM therapies along with their conventional therapy	100 (41.3)	82 (33.9)	42 (17.4)	12 (5.0)	6 (2.5)
The medical professionals should encourage referrals to CAM therapy and make it available to the patient along with conventional therapy	22 (9.1)	72 (29.8)	87 (36.0)	45 (18.6)	16 (6.6)
The medical curriculum should include courses about CAM along with practical training	19 (7.9)	64 (26.4)	79 (32.6)	46 (19.0)	34 (14.0)

**Table 5:** Senior medical students' attitude towards CAM.

Students' characteristics	Complementary and alternative medicine attitude score percentage			*p* value
	Median	IQR	Mean rank	
Age (years)				
≤22 (*n* = 106)	66.0	58.0–74.0	110.3	0.027^*∗*^
>22 (*n* = 136)	70.0	62.5–74.0	130.2	
Gender				
Males (*n* = 125)	68.0	60.0–74.0	117.0	0.294^*∗*^
Females (*n* = 117)	68.0	62.0–74.0	126.1	
Academic year				
4th (*n* = 96)	66.0	58.0–72.0	101.7	<0.001^*∗∗*^
5th (*n* = 60)	68.0	60.5–74.0	123.4	
6th (*n* = 86)	70.0	64.0–74.5	142.3	

^*∗*^Mann-Whitney test. ^*∗∗*^Kruskal-Wallis test.
